# Temperature Requirements Can Affect the Microbial Composition Causing Sour Rot in Grapes

**DOI:** 10.1111/1758-2229.70061

**Published:** 2025-01-27

**Authors:** Chiara Brischetto, Vittorio Rossi, Irene Salotti, Luca Languasco, Giorgia Fedele

**Affiliations:** ^1^ Department of Sustainable Crop Production (DI.PRO.VE.S.) Università Cattolica del Sacro Cuore Piacenza Italy; ^2^ Research Center on Plant Health Modelling (PHeM) Università Cattolica del Sacro Cuore Piacenza Italy

**Keywords:** bioclimatic characters, bunch microflora, controlled environment, microclimate, minor bunch rot, optical density, *Vitis vinifera*

## Abstract

Sour rot (SR) is a late‐season non‐Botrytis rot affecting grapevines, resulting from a complex interplay of microorganisms, including non‐*Saccharomyces* yeasts and acetic acid bacteria. Nonmicrobial factors contributing to disease development encompass vectors (e.g., *Drosophila* spp.), the presence of wounds or microcracks on grape berry surfaces, and environmental conditions during berry ripening. The microbial complexes within SR‐affected grapes exhibit variability among different bioclimates and seasons, with certain microorganisms predominating under specific conditions. This study examined the influence of environmental conditions on the microbiome composition associated with SR‐affected grape bunches, utilising data from 41 locations across three distinct Italian bioclimates. We selected nine yeast and two bacterial species frequently isolated from sour‐rotted grapes for analysis. The growth responses of these microorganisms to temperature were assessed by categorising them into four ecophysiological clusters. Furthermore, we analysed the distribution of these microorganisms and their respective ecophysiological clusters across the three bioclimates. The results indicate that the microbiomes involved in SR can vary according to the bioclimatic conditions of the grape‐growing area. Further research is required to comprehend the ecological requirements of these microorganisms, define their ecological niches to understand their geographical distribution and epidemiology, and enhance SR management strategies.

## Graphical abstract

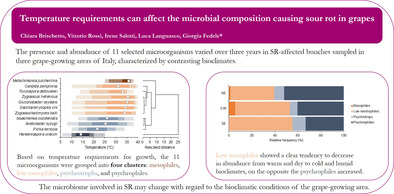

## 1 Introduction

Sour rot (SR) of grapes is a late‐season bunch rot caused by a complex of microorganisms (Guerzoni and Marchetti 1987; Gravot et al. 2001; Barata, Malfeito‐Ferreira, and Loureiro 2012). Non‐*Saccharomyces* yeasts (NSYs) and acetic acid bacteria (AAB) were the most frequently isolated microorganisms from the affected berries, based on a recent systematic literature review (Brischetto, Rossi, and Fedele 2024a). Indeed, these microorganisms are considered the most important components of the microbial complex causing SR. These microorganisms usually present a metabolic‐based temporal succession. SR develops during berry ripening due to the increasing sugar that becomes available for the metabolism of the microbial population (Gravot et al. 2001; Marchetti, Guerzoni, and Gentile 1984). In the early stage of SR, a low glucose concentration promotes NSY‐producing ethanol. Then, AABs could take advantage of the oxidation of ethanol into acetic acid, becoming the dominant microbial SR population during the late stages of the process (Pinto et al. 2019).

However, the presence of these microorganisms in SR‐affected grapes varies among grape‐growing areas and seasons, with some of them prevalent in some situations but not in others. For instance, *Acetobacter* was found prevalent in SR‐affected bunches in grape samples collected in Geneva (NY) in 2015 (Hall et al. 2019), in coastal eastern China (Gao et al. 2020), in the Adelaide Hills region of Australia in 2011 (Mateo et al. 2014), in Lisbon, Portugal, in 2007 (Barata et al. 2012), but not in samples from Tasmania, Australia, in 2016 (Hall et al. 2019) or Italian samples from 2019 to 2021 (Brischetto, Rossi, and Fedele 2024b). Changes in the SR microbial complex based on geography suggest that nonmicrobial factors are related to the disease. The presence of *Drosophila* and the wounding of the grape berry surface are among these factors (Hall et al. 2019). Environmental conditions during berry ripening may also play a role, but they have not been investigated in deep (Brischetto, Rossi, and Fedele 2024a).

Some studies have reported that warm and rainy weather between veraison and harvest were conducive to the SR epidemic (Zoecklein, Williams, and Duncan 2000; Huber 2016), but the specific weather conditions in which the disease developed have not been reported. It is commonly accepted that SR develops rapidly and severely at temperatures between 20°C and 25°C, moderately at temperatures between 15°C and 20°C and barely develops at temperatures between 10°C and15°C (McFadden‐Smith and Gubler 2015). Accordingly, SR was severe in three seasons, where the temperature ranged between 8°C and 24°C during disease development in Ontario (Canada), but not in the fourth season when the temperature was between 4°C and 15°C (Huber 2016). However, the SR incidences observed in the two seasons were more related to hours with temperatures above 30°C or days with maximum temperatures above 30°C than other meteorological variables measured in Catalonia (Spain) (Calvo‐Garrido et al. 2013). The literature information is then contrasting and inconclusive.

The study of temperature‐dependent growth patterns of SR‐associated microorganisms would provide useful information. Unfortunately, there is only one study on the effect of temperature (5°C–10°C, 10°C–15°C, 15°C–20°C and 20°C–25°C) on SR development in wounded berries artificially inoculated with *Hanseniaspora uvarum* and *Gluconobacter oxydans*, as far as we know. In the 7 days following inoculation, disease severity increased at increasing temperatures, with the most severe disease occurring at 20°C–25°C and low disease at < 10°C for both microorganisms (Huber 2016).

In this research, we aimed to investigate the effect of environmental conditions on the composition of the microbiome associated with grape bunches affected by SR. To achieve this aim, we (i) investigated the microbiome related to SR‐affected bunches across 41 locations in three contrasting bioclimates of Italy over 3 years. The bacterial and fungal microbiota affected (i.e., exhibiting visual and olfactory SR symptoms) ripe bunches were determined using sequencing and quantitative PCR (qPCR) to assess the relative abundance and changes of 11 key microorganisms associated with SR, including yeasts and bacteria. Afterward, we (ii) investigated the effect of temperature on the growth of representative strains of the above microorganisms and grouped them into four ecophysiological clusters. Finally, we (iii) analysed the distribution of microorganisms and their ecophysiological clusters in the three contrasting bioclimates.

## 2 Materials and Methods

### 2.1 Sample Collection and Microbiome Analysis

Grape samples were collected from vineyards grown in 41 locations in three grape‐growing areas of Italy in 2019, 2020 and 2021. These areas are characterised by contrasting bioclimates, as described in Table 1. We named these bioclimates based on their geography as south‐east (SE), central‐west (CW) and north‐east (NE). In short, SE and CW are warmer than NE, and CW and NE are more humid than SE. The three bioclimates differ in thermotype, ombrotype and continentality (Table 1).

**TABLE 1:** The main climatic and bioclimatic characteristics of the three grape‐growing areas of Italy considered in the study.

Characters	South‐east (SE)	Central‐west (CW)	North‐east (NE)	Reference
Regions	Apulia	Tuscany and Lazio	Emilia‐Romagna and Friuli‐Venezia Giulia	—
Köppen–Geiger climate^a^	Cfa	Csa	Cfa	Peel et al. 2007
Temperature^b^	14°C–16°C	14°C–16°C	13°C–14°C	Costantini et al. 2013
Rainfall^c^	400–700 mm	700–1000 mm	700–1000 mm	
Aridity index^d^	Dry sub‐humid	Sub‐humid	Sub‐humid	
Bioclimate and variants^e^	Mediteranean	Mediterranean	Temperate, Stp and Sbm variants	Pesaresi et al. 2014
Thermotype^f^	Lower mesa Mediterranean	Upper meso Mediterranean	Upper meso temperate	
Ombrotype^g^	Upper dry	Lower dry	Upper subhumid	
Continentality^h^	Weak oceanic	Weak semicontinental	Strong semicontinental	

^a^ Cfa: temperate, no dry season, hot summer; Csa: temperate, dry summer, hot summer.

^b^ Mean annual air temperature.

^c^ Total annual rain.

^d^ Based on mean annual precipitation and annual reference evapotranspiration (UNEP, 1997).

^e^ Stp: steppic; Sbm: submediterranean.

^f^ Based on the compensated thermicity index and positive temperature threshold values (Rivas‐Martínez et al. 2011).

^g^ Based on the ombrothermic index, which is calculated as a function of both the total positive precipitation and temperature.

^h^ Based on the difference between the highest and lowest monthly average temperatures of the year.

We collected 15 random ripe bunches affected by SR (i.e., exhibiting visual and olfactory SR symptoms) in each vineyard and transported them to the laboratory. From each cluster, 100 berries were randomly removed with sterilised scissors and then manually pressed in a plastic bag. The obtained must (100 mL) was extracted and placed into two 50 mL Falcone tubes. The samples were stored at −20°C until molecular analysis. The samples were sent to WineSeq laboratories (https://www.wineseq.com; https://www.biomemakers.com), and total DNA extraction and next generation sequencing (NGS) of the samples were performed as described by (Brischetto, Rossi, and Fedele 2024b).

Data were analysed using MicrobiomeAnalyst 2.0, specifically the community profiling, correlation network and comparison functionalities (https://www.microbiomeanalyst.ca). The effect of bioclimates on the distribution and abundance of the 11 microorganisms was evaluated by considering alpha diversity to measure the diversity with a sample using the Shannon index, which accounts for both richness and evenness of operational taxonomic units (OTUs), and the Chao1 index, the post hoc pairwise comparison was performed with the Mann–Whitney test. Single‐factor statistical comparisons were made using EdgeR, a method that uses the negative binomial distribution to model read count data with replicates and generalised linear models for pairwise comparisons between groups of samples in a dataset. The correlation network analysis was performed using sparse estimation of correlations among Microbiomes (SECOM) for estimating linear and nonlinear relationships among pairs of microorganisms across bioclimates while maintaining sparsity.

### 2.2 Temperature‐Dependent Growth of Microorganisms

Nine yeasts and two bacteria species (Table 2) were selected, frequently isolated from rotten berries, and putatively involved in SR (Brischetto, Rossi, and Fedele 2024a). Yeasts were grown onto GYP liquid media with 2% glucose, 0.5% yeast extract, 0.5% peptone and 2% agar (Carlo Erba Reagents) at 25°C for 48 h. In comparison, bacteria were grown onto YPM liquid media with 0.5% yeast extract, 0.3% peptone, 2.5% D‐mannitol and 2% agar (Carlo Erba Reagents) for 48 h at 25°C. For long‐term storage, isolates were frozen at −20°C in vials containing GYP or YPM broth with 50% (v/v) glycerol.

**TABLE 2:** List of microorganisms used.

Microorganism	Strain	Isolated from
*Starmerella bacillaris* (formerly *Candida zemplinina*)	CBS 9494	Wine (HU)
*Metschnikowia pulcherrima*	CBS 4873	Grape (DE)
*Hanseniaspora uvarum*	CBS 8130	Grapes affected by SR (IT)
*Pichia terricola* (syn. *Issatchenkia terricola*)	CBS 8131	Grapes affected by SR (IT)
*Zygoascus hellenicus* (syn. *Candida steatolytica*)	CBS 6736	Efflue (JP)
*Issatchenkia occidentalis* (syn. *Pichia occidentalis*)	CBS 10322	Grape juice (AU)
*Zygosaccharomyces bailii*	CBS 4688	Grape must (IT)
*Saccharomycopsis vini* (syn. *Endomycopsella vini*)	CBS 4097	Grape must (BR)
*Torulospora delbrueckii*	CBS 1151	Grape must (IT)
*Gluconobacter oxydans*	LMG 1408	Beer (unknown)
*Acetobacter syzygii*	LMG 21419	*Syzygium malaccense* (ID)

Microorganisms of Table 2 were grown in a liquid substrate mimicking the chemical composition of ripened grape berries (herein named GS89 from the growth stage classification of Lorenz et al. 1995), which was prepared as described by Ciliberti et al. (2016) by adding sugars (12.175% glucose and 12.825% fructose; Carlo Erba Reagents), organic acids (0.25% malic and 0.25% tartaric acid; Carlo Erba Reagents) and salts (6.7% ammonium sulphate, 6.7% ammonium dihydrogen phosphate, 15% monopotassium phosphate and 7.5% magnesium sulphate; Carlo Erba Reagents) to double distilled water. The pH of the substrate was adjusted to 3.5 using potassium hydroxide or phosphoric acid (Carlo Erba Reagents) after autoclaving.

The artificial medium GS89 was inoculated with a suspension of each microorganism in 10 mL Falcon tubes (1:9, v/v) and vortexed for 20 s. The tubes were then incubated at 5°C, 10°C, 15°C, 20°C, 25°C, 30°C, 35°C or 40°C in a growth chamber for 7 days, 100% relative humidity, and 12 h photoperiod. The temperature range 5°C–40°C was selected based on previous study on both the effect of temperature on SR development (Huber 2016) and the average temperature conditions in vineyards during the grape ripening stages. There were three tubes (replicates) for each combination microorganism × temperature. Three aliquots of 100 μL of the suspension (stock suspension) were then taken from each tube and loaded into 96‐well plates. Non‐inoculated artificial substrates were used to measure substrate turbidity (control). The plates were shaken at 567 cpm linear frequency for 30 s before every automatic reading. The suspension turbidity was measured using a BioTek 800 TS absorbance reader (Agilent Technologies, Santa Clara, CA 95051, USA) as the optical density (OD) using a 620 nm filter (OD_620_) and Gen5 e provided by the manufacturer. The experiment was performed twice. The OD data were then used to estimate the colony forming units (CFUs) present in the suspension through calibration equations, which have been generated as follows.

The stock solution was diluted in the following proportions: 1:2.5, 1:5, 1:7.5 and 1:10. A 100 μL of the initial suspension of each dilution was loaded in 96‐well plates, each in three replicates. A negative control consisting of non‐inoculated 100 μL of GS89 medium was considered. A total of 100 μL of the last dilution (1:10) for each yeast/bacterial suspension were further 10‐, 100‐ and 1000‐fold diluted, and each dilution was plated on GYP/YPM solid media in Petri plates (9 cm in diameter); there were three plates (replicates) per each combination microorganism × dilution. Plates were incubated at 25°C for 48 h, 100% relative humidity, and a 12 h photoperiod. Afterward, the CFUs were enumerated. The calibration experiment was performed twice. For the development of the calibration equations, the turbidity data were corrected for the turbidity of the negative control. They were regressed against the corresponding CFUs using the *lm* function of the R ‘stats’ package (version 4.3.1; R Core Team, 2023). The results are shown in Table S1. Since no clear relationship was found between OD data and CFUs for *H. uvarum* and *P. terricola* , the CFUs at different temperatures were enumerated through plate counting.

CFU data were first rescaled to the highest number found in the experiment (at the optimal temperature for growth) to study the relationship between temperature and CFUs. Rescaled data were then regressed against temperature using different nonlinear regression equations. The best equations were selected based on Akaike's information criterion (AIC). The equations of Analytis (1977) and Duthie (1997) provided the smallest AIC values, depending on the microorganisms, and were therefore considered the most likely to be correct (Burnham and Anderson, 2002).

The Analytis (1977) equation was used in the following form:

(1) $$y={\left[a\times {\mathrm{Teq}}^{b}\times \left(1—\mathrm{Teq}\right)\right]}^{c}$$

where *y* is the rescaled CFUs (on a 0–1 scale); Teq is the temperature equivalent, defined as Teq = (T − Tmin)/(Tmax−Tmin), where T is the temperature regime (in °C) and Tmin and Tmax are minimal and maximal temperatures for microorganism growth (in °C); a–c are the equation parameters, with a, b and c defining the top, symmetry and size of the bell‐shaped curve, respectively.

The Duthie (1997) equation was used as follows:

(2) $$Y=E^{'}\left\{\exp.\left[\left(T—f\right)g/\left(h+1\right)\right]\right\}/\left\{1+\exp.\left[\left(T—f\right)g\right]\right\}$$

where E' = E[(h + 1)/h]h^1/(h + 1)^ and T is the temperature regime (in °C). This equation defines a unimodal curve where the response declines from 1, the maximum value for *y*, and approaches a lower limit of 0 as the temperature increases or decreases from the optimum. The intrinsic rate of decline and the degree of asymmetry in temperature response are described by the parameters g and h, respectively.

The equation parameters Tmin, Tmax, and a–c in Equation (1); f, g in Equation (2) were estimated using the function *nls* of the ‘stats’ package. The goodness‐of‐fit was estimated based on the adjusted *R* ^2^, the root mean square error (RMSE), the coefficient of residual mass (CRM), and the concordance correlation coefficient (CCC) (Nash and Sutcliffe 1970; Lin, 1989). In brief, the RMSE is the average distance of real data from the fitted line and was obtained using the *rmse* function of the R ‘modelr’ package (Wickham 2019). CRM is a measure of the tendency of the equation to overestimate or underestimate the observed values (a negative CRM indicates a tendency of the model towards overestimation) (Nash and Sutcliffe 1970). CCC is the product of two terms: the Pearson product–moment correlation coefficient between observed and predicted values and the coefficient Cb, which indicates the difference between the best fitting line and the perfect agreement line (if CCC = 1, the agreement is perfect (Lin, 1989)). The CCC was obtained using the CCC function of the R ‘DescTools’ package (Signorell 2020). The distribution of residuals (i.e., observed‐fitted values) was also analysed. The optimal temperature (Topt) for microorganism growth was calculated as Topt = [(b × c)/(b × c + c)] × (Tmax—Tmin) + Tmin, for Equation (1); and Topt = f–(1/g) ln(h), for Equation (2).

### 2.3 Ecophysiological Traits of Microorganisms

Based on the effect of temperature on colony growth, the microorganisms were grouped into ecophysiological groups (McMeekin et al. 2010) using hierarchical cluster analysis. Data for multivariate clustering were obtained from Equations (1) and (2), specifically: Tmin, Tmax and Topt, temperature range at which the microorganisms had moderate (between 10% and 50% of maximal growth), sustained (between 50% and 80%) and optimal (more than 80%) growth. Agglomerative clustering was based on the between‐groups linkage using squared Euclidean distance. Clusters were then assigned to one of the following types (Precht 2013): mesophiles, psychrotrops and psychrophiles.

### 2.4 Analysis of the Microbiome in the Three Bioclimatic Areas

For each OTU, the median distribution of the reads in each bioclimate and year was first calculated and expressed as the relative frequency of the total OTUs in that bioclimate and year. These relative frequencies were then averaged over the three bioclimates for single microorganisms and their ecophysiological clusters.

## 3 Results

### 3.1 Abundance and Frequency of SR‐Associated Microorganisms

**FIGURE 1:**
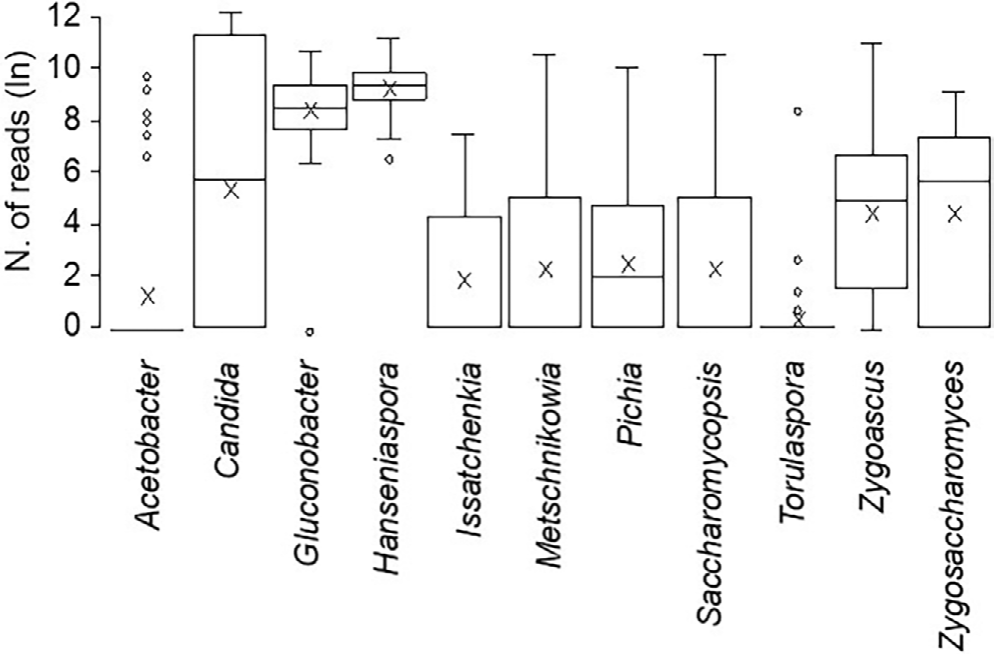
Box plots showing the abundance (expressed as the natural logarithm of the reads) of 11 microorganisms associated with grape bunches affected by sour rot, collected in 41 vineyards in three Italian grape‐growing regions from 2019 to 2021. Box plots extend from the 25th to the 75th quartile of the data distribution. The lines crossing the boxes represent the median, and × indicates the average. Whiskers extend to the maximum and minimum, and white dots indicate outliers.

**FIGURE 2:**
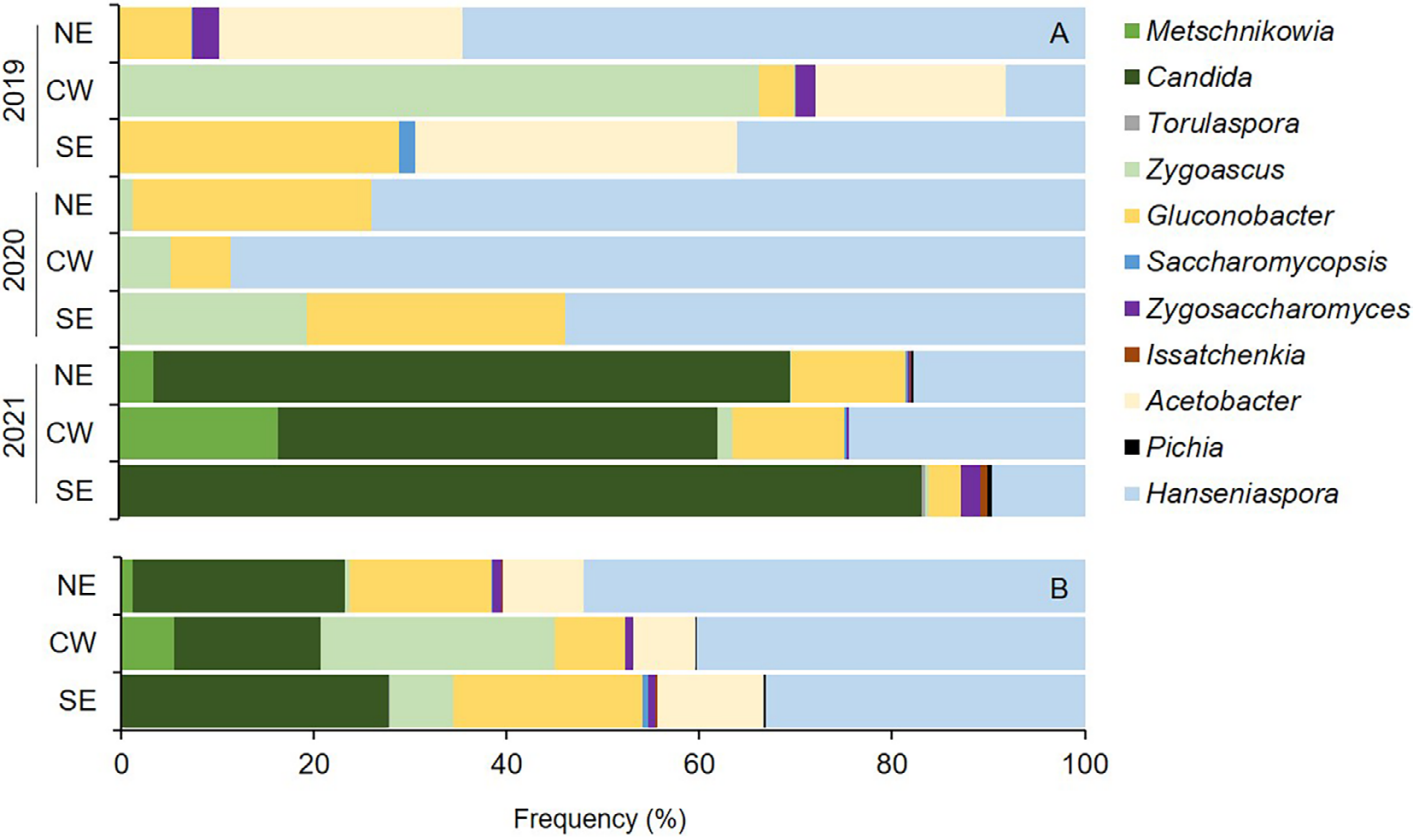
Relative frequency of 11 microorganisms associated with grape bunches affected by sour rot in three bioclimates of Italy (south‐east, SE; central‐west, CW; north‐east, NE; see Table 1) in three grape‐growing seasons (A) and the average of seasons (B).

**FIGURE 3:**
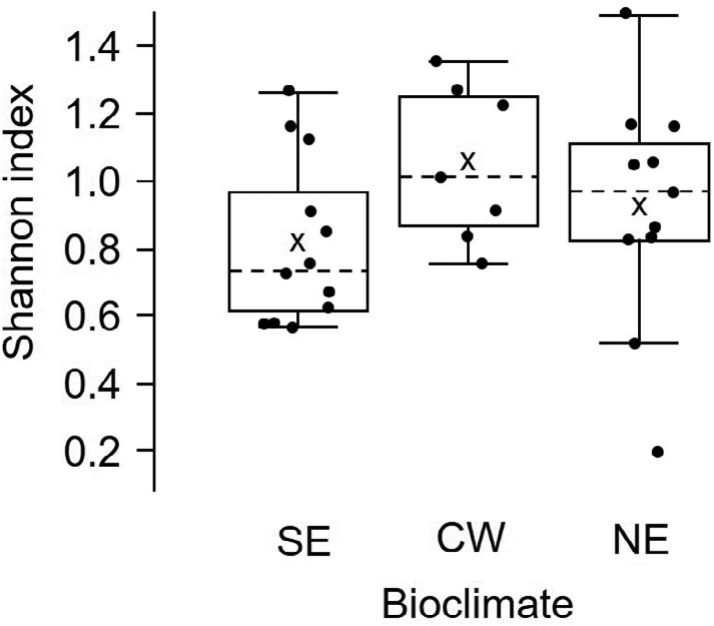
Box plots showing the alpha diversity (measured using the Shannon index) of 11 microorganisms associated with grape bunches affected by sour rot, which were collected in 41 vineyards in three grape‐growing areas that represent different bioclimates of Italy from 2019 to 2021. Bioclimates are defined in Table 1: South‐east, SE; central‐west, CW; north‐east, NE. Box plots extend from the 25th to the 75th quartile of the data distribution. The dotted lines crossing the boxes represent the median, and × indicates the average; whiskers extend to the maximum and minimum, and black dots indicate the Shannon index value for each sample.

The abundance of the 11 microorganisms in the grape samples is shown in Figure 1. The relative frequencies of the 11 microorganisms in different years and bioclimates are shown in Figure 2. The AAB *Gluconobacter* and the NSY *Hanseniaspora* were the prevalent microorganisms, with slight variability among the samples. *Acetobacter* and *Torulaspora* were the less abundant microorganisms, with some outliers. *Candida*, *Zygoascus* and *Zygosaccharomyces* were abundant, with high among‐sample variability. Some species showed different frequencies concerning bioclimates. Among the most abundant microorganisms, *Candida* was more frequent in the SE (27.7% of total microorganisms) than in the CW (15.1%), and *Zygoascus* was more abundant in the CW (24.4%) than in the NE (0.5%). In comparison, *Hanseniaspora* progressively increased from SE (33.2%) to CW (40.3%) and NE (52.0%). *Gluconobacter* and *Acetobacter* did not show evident differences in their frequency. Overall, microbial diversity was affected by the bioclimate for both richness and evenness of microorganisms, with a *p*‐value of alpha‐diversity equal to 0.132, with the SE bioclimate having a Shannon index lower than CW, with *p* = 0.036 (Figure 3).

### 3.2 Temperature‐Dependent Growth of SR‐Associated Microorganisms

**FIGURE 4:**
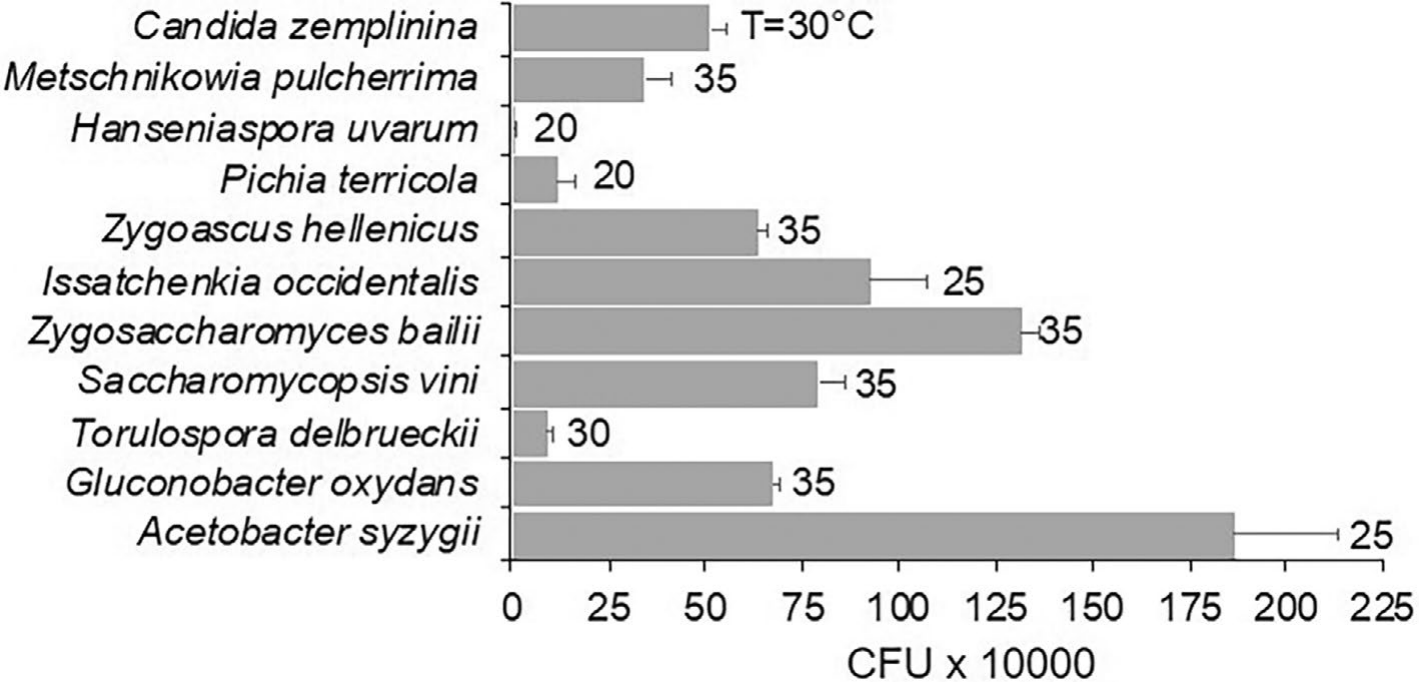
Number of colony forming units (CFUs) of 11 microorganisms associated with grape bunches affected by sour rot, which have grown for 7 days in a liquid medium mimicking ripened grapevine berries. Bars and whiskers are the averages and standard errors of three replicates. The numbers on the right of the bars show the incubation temperature with the highest CFUs (colonies were incubated at 5°C, 10°C, 15°C, 20°C, 25°C, 30°C, 35°C or 40°C).

The different microorganisms showed different growth capabilities on the medium, mimicking ripened grapevine berries (Figure 4). *H. uvarum* , *T. delbrueckii*, and *P. terricola* produced 8.22 × 10^4^, 9.32 × 10^4^ and 1.18 × 10^6^ CFU after 7 days of incubation at 20°C, 30°C and 20°C, respectively. At the same time, *A. syzygii* produced 1.86 × 10^7^ CFU at 25°C. The other microorganisms showed intermediate growth.

**FIGURE 5:**
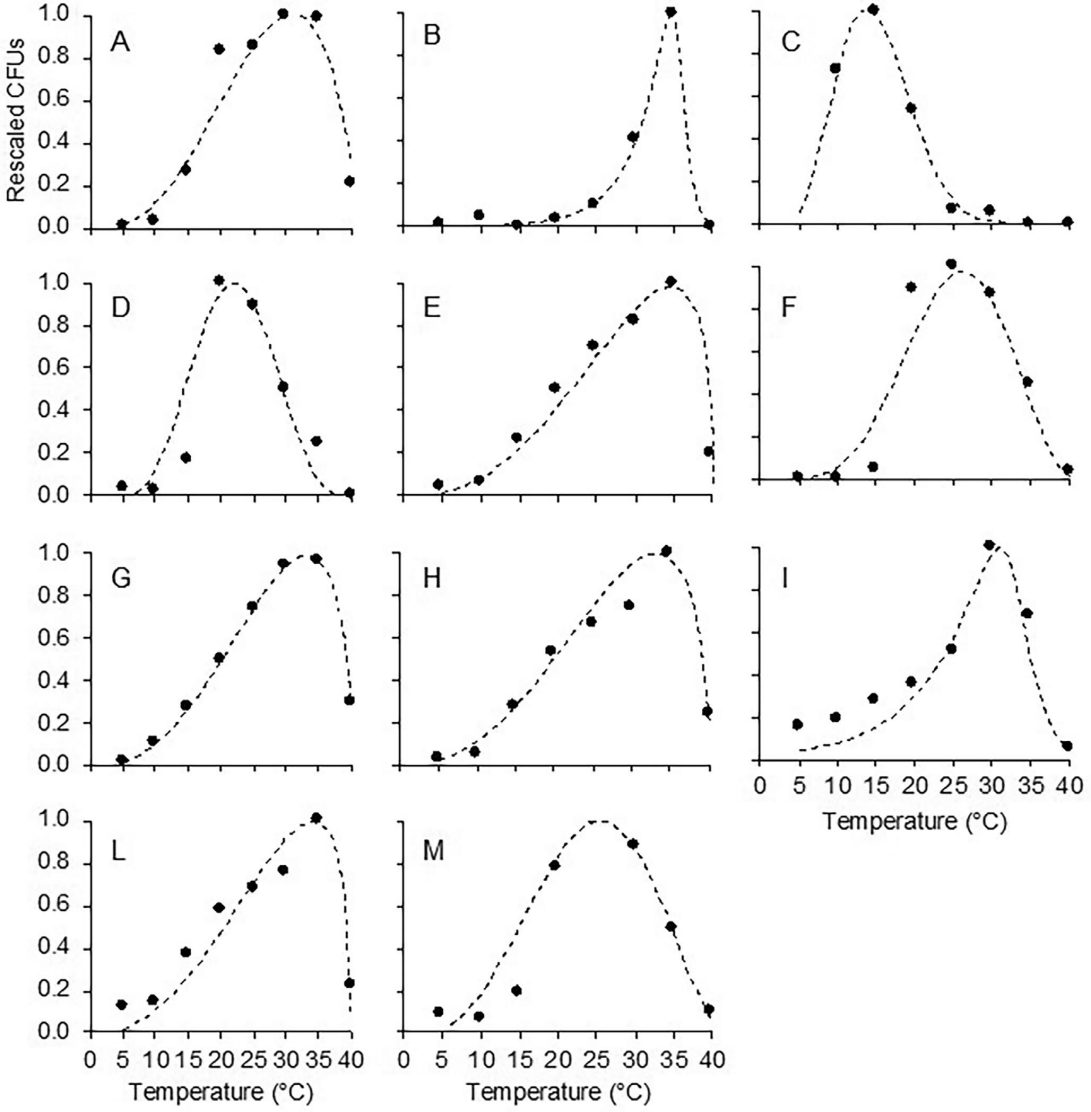
Effect of temperature on the rescaled number of colony forming units (CFUs) of (A) *Candida zemplinina*, (B) *Metschnikowia pulcherrima*, (C) *H. uvarum* , (D) *Pichia terricola* , (E) *Zygoascus hellenicus*, (F) *Issatchenkia occidentalis*, (G) *Zygosaccharomyces bailii*, (H) *Saccharomycopsis vini*, (I) *Torulospora delbrueckii*, (L) *Gluconobacter oxydans* and (M) *Acetobacter syzygii* . Microorganisms were grown on an artificial medium mimicking the composition of ripe berries at different temperatures between 5°C and 40°C. Dots are the averages of three replicates. Lines fit the data based on Equations (1) and (2) with the parameters listed in Table 3.

The different microorganisms also showed different temperature‐response patterns (Figure 5). Cardinal temperatures, estimates of equation parameters with their standard errors, and goodness‐of‐fit of Equations (1) and (2) are reported in Table 3. Overall, the two equations provided a good fit for the rescaled number of CFUs at different temperatures for any microorganism, with *R* ^2^ > 0.8, CCC ≥ 0.912, RMSE ≤ 0.155, and CRM between −0.101 and 0.173 (Table 3).

**TABLE 3:** Parameters and statistics of the equations fitting the temperature response of microorganisms related to the sour rot of grapes.

Microorganism	Tmin (°C)	Topt (°C)	Tmax (°C)	Equation parameters^a^			Statistics of goodness of fit			
				a/f	b/g	c/h	*R* ^b^	CCC	RMSE	CRM
*C. zemplinina* ^c^	2.0	31.0	41.0	2.149	2.917	0.822	0.943	0.965	0.100	0.013
				0.161	0.427	0.222				
*M. pulcherrima* ^b^	5.0	35.8	41.0	35.829	1.387	4.769	0.996	0.998	0.019	0.014
				0.291	0.383	1.966				
*H. uvarum* ^c^	2.0	14.2	40.0	7.342	0.453	8.844	0.994	0.997	0.026	0.012
				0.151	0.011	0.784				
*P. terricola* ^c^	4.0	21.5	40.0	4.000	1.000	3.500	0.808	0.912	0.155	−0.067
				0.438	0.144	1.448				
*Z. hellenicus* ^c^	1.0	35.4	40.2	1.600	6.000	0.400	0.942	0.974	0.076	−0.012
				0.084	0.922	0.088				
*I. occidentalis* ^c^	1.0	26.0	42.0	2.940	1.600	2.981	0.913	0.951	0.122	0.031
				0.208	0.175	0.957				
*Z. bailii* ^c^	1.0	33.9	40.3	1.772	4.518	0.528	0.981	0.991	0.045	−0.001
				0.058	0.375	0.068				
*S. vini* ^c^	1.0	34.2	40.1	1.750	4.700	0.490	0.951	0.974	0.078	−0.043
				0.108	0.779	0.125				
*T. delbrueckii* ^b^	0	33.0	42.0	33.000	0.629	3.756	0.925	0.941	0.104	0.173
				0.719	0.074	0.986				
*G. oxydan* ^c^	1.0	35.1	40.0	1.646	5.640	0.390	0.942	0.945	0.113	0.090
				0.368	4.041	0.435				
*A. syzygii* ^c^	1.0	25.3	42.0	3.100	1.478	2.050	0.887	0.942	0.123	−0.101
				0.260	0.181	0.644				

*Note:* Microorganisms were grown on an artificial medium mimicking the composition of ripe berries at different temperatures between 5°C and 40°C, and the growth was expressed as colony forming units (CFUs).

^a^ a, b and c are parameters of Equation (1); f, g and h for Equation (2); numbers in italics are standard errors of parameter estimates.

^b^ Fit with Equation (2).

^c^ Fit with Equation (1).

**FIGURE 6:**
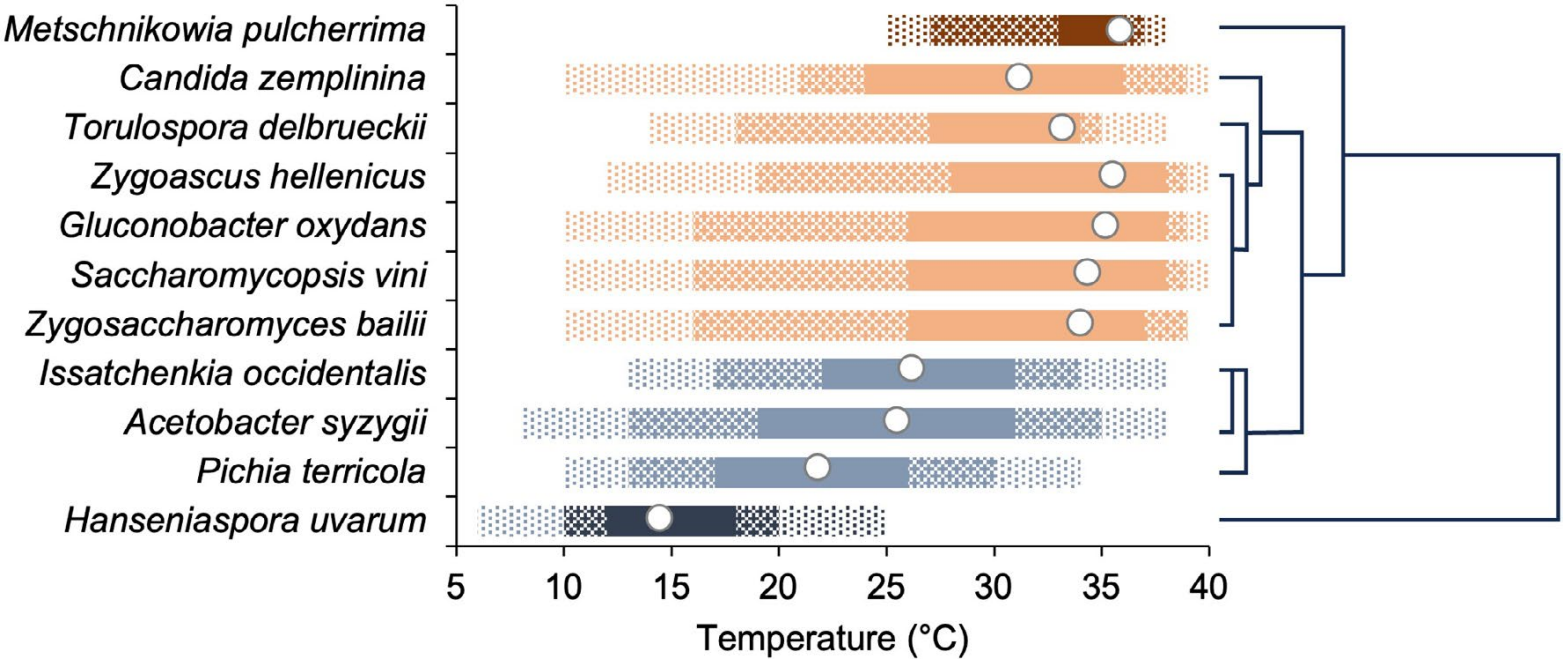
Temperature range for moderate (between 10% and 50% of maximal growth; dotted bars), sustained (between 50% and 80%; grid bars) and optimal (more than 80%; full bars) growth, and the optimal temperature (white dots) of 11 microorganisms associated with grape bunches affected by sour rot, based on the growth curves of Figure 3. The dendrogram identifies four clusters of microorganisms: Mesophiles (brown bars), low mesophiles (orange), psychrotrops (light blue) and psychrophiles (blue) based on the rescaled distance for cluster combine (using average linkage between groups).

Figure 6 shows the temperature range at which the microorganisms had moderate, sustained, and optimal growth and the optimal temperature based on the growth curves of Figure 5. The hierarchical cluster analysis distinguished four groups (Figure 6). Group I included only *M. pulcherrima* , with sustained growth at 31°C–37°C, and Topt at 35.5°C, which can be considered a mesophile microorganism. Group II included *Z. bailii*, *S. vini*, *Z. hellenicus*, *T. delbrueckii*, *C. zemplinina* and the AAB *G. oxydans* , with sustained growth at 20°C to < 40°C, and Topt at the 30°C–35°C range, which can be considered as low mesophiles. Among this group, *C. zemplinina* showed the widest temperature range for growth. Group III included *P. terricola* , *I. occidentalis* and *A. syzygii* with optimal growth at > 15°C to approximately 30°C and Topt at 20°C to 25°C/26°C, which can be considered psychrotrophs. Finally, Group IV included only *H. uvarum* , with sustained growth at 8°C–20°C, and Topt at 14.2°C, considered a psychrophile.

### 3.3 Relationships Between Ecophysiological Groups and Bioclimates

**FIGURE 7:**
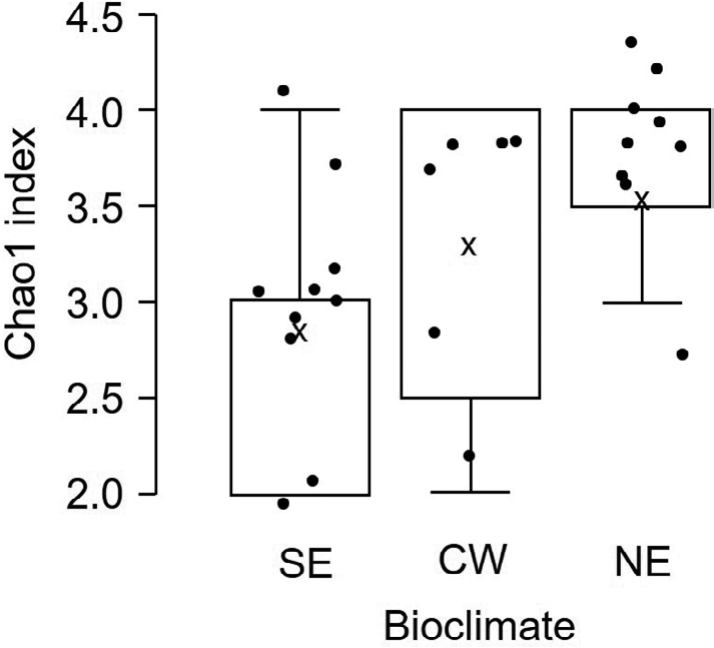
Box plots showing the alpha diversity (measured using the Chao1 index) of 11 microorganisms associated with grape bunches affected by sour rot, which were collected in 41 vineyards in three grape‐growing areas representing different Italy bioclimates from 2019 to 2021. Microorganisms were aggregated into four ecophysiological groups, as indicated in Figure 6. Bioclimates are defined in Table 1: South‐east, SE; central‐west, CW; north‐east, NE. Box plots extend from the 25th to the 75th quartile of the data distribution. The black dots indicate the Chao1 index value for each sample, and × indicates the average; whiskers extend to the maximum and minimum.

**FIGURE 8:**
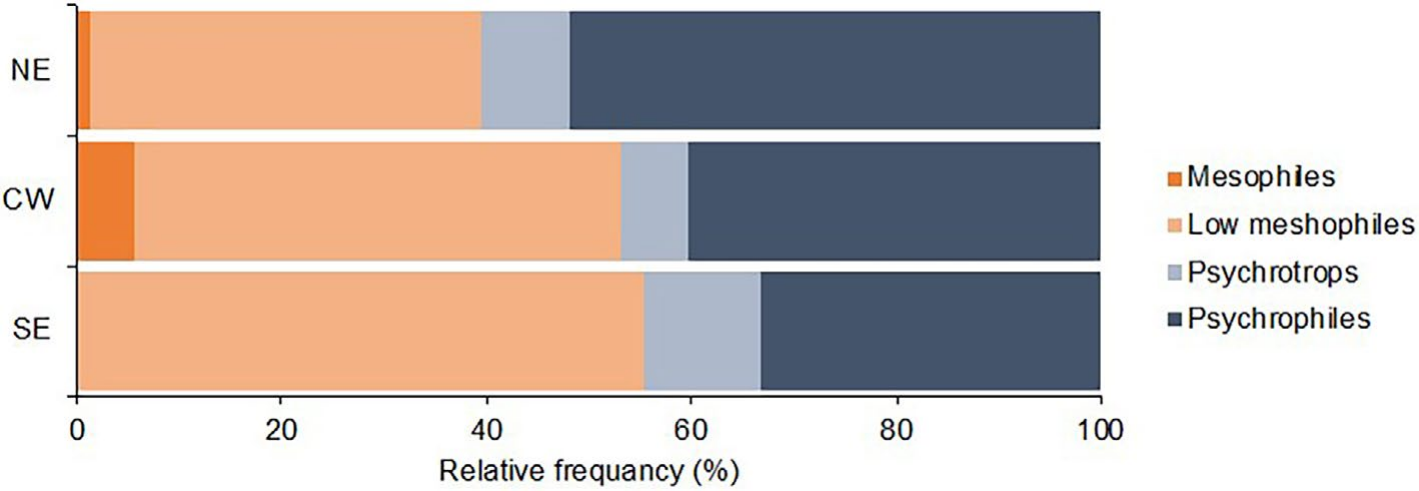
Relative frequency of four ecophysiological groups of microorganisms associated with grape bunches affected by sour rot. Groups were defined based on temperature requirements from growth, as shown in Figure 6.

The alpha diversity showed increasing values of the Chao1 index from SE, CW and NE (*p* = 0.089), with a significant difference between SE and NE (*p* = 0.032) (Figure 7) when the microorganisms in the three bioclimates were aggregated based on their belonging to the four clusters of temperature response (see Figure 6). Concerning the frequency (Figure 8), there was a clear tendency of low mesophiles to decrease in abundance from SE (55.3% of total microorganisms) to CW (46.6%) and NE (38.3%) and of psychrophiles (i.e., *Hanseniaspora*) to increase (33.2%, 40.3% and 52.0%, in the three bioclimates, respectively). The abundance of these two groups was negatively correlated, with a correlation coefficient = −0.714 (*p* < 0.001). Psychrotrophs did not show a clear trend, while mesophiles (including only *Metschnikowia*) were more abundant in CW.

## 4 Discussion

We selected nine yeasts and two bacteria species in this research that are often isolated from sour rotten grapes and considered essential components of the microbial consortia causing grape SR (Brischetto, Rossi, and Fedele 2024a). These microorganisms included yeasts with low fermentative activity (i.e., *Hanseniaspora*), aerobic yeasts (*Pichia*, *Candida* and *Metschnikowia*), yeasts with fermentative metabolism (*Torulaspora* and *Zygosaccharomyces*), a lipolytic yeast (*Saccharomycopsis*) and the AAB *Gluconobacter* and *Acetobacter* (Jolly, Augustyn, and Pretorius 2006; Barata, Malfeito‐Ferreira, and Loureiro 2012; Guerzoni and Marchetti 1987).

We found that the presence and abundance of these microorganisms varied over 3 years in SR‐affected bunches sampled at 41 locations in three grape‐growing areas of Italy, which are characterised by contrasting bioclimates. We used a bioclimatological approach (mainly based on temperature and precipitation patterns) for describing the three areas because it is highly informative for studying the relationship between climate and the distribution of living organisms and their communities (Rivas‐Martínez et al. 2011), including microbial populations (Costa et al. 2022; Picazo et al. 2020; Yang et al. 2022). The three bioclimates belong to different thermotypes and ombrotypes, and have different continentality indexes, with a gradient from warm and dry to mild and humid moving from SE, CW and NE bioclimates.

To understand whether the above differences were related to the temperature requirements of the different microorganisms, we studied colony growth at a 5°C–40°C range of SR‐relevant species (one strain for each species). We know that temperature requirements may vary among individuals belonging to the same genus and species, and this variability could affect our results somewhat. However, the impact of this variability remains to be explored. For instance, the Bacterial Diversity Metadatabase (BacDive; https://bacdive.dsmz.de)—the largest worldwide database for standardised bacterial phenotypic information—contains information on the temperature‐dependent growth of strains of five *Acetobacter* spp. related to grapes, that is, *A. aceti* (nine entries), *A. cerevisiae* (three), *A. malorum* (one), *A. musti* (one) and *A. syzygii* (one). For all these species, there was no growth at 10°C or 37°C–45°C, and growth at 10°C–37°C. Similarly, all 26 entries of *G. oxydans* were reported to grow actively at 25°C–30°C, showing inconsistent growth at 33°C–34°C and no growth at 5°C–10°C or 35°C–37°C. In *Saccharomyces cerevisae*, the optimal temperature of different strains of fermenting wines was similar (ranging between 31.3°C and 32.1°C), while strains of different origins may diverge (Salvadó et al. 2011). Further studies are needed to study the variability in the temperature response of the species and strains of SR‐associated microorganisms.

Since the chemical composition of the medium affects microbial population growth, we used a liquid substrate mimicking the chemical composition of ripened grapevine berries, which has already been used for *B. cinerea* and some biocontrol agents, including yeast‐ and bacteria‐based products (Ciliberti et al. 2016; Altieri, Rossi, and Fedele 2023). The use of an artificial substrate instead of field‐collected berries has the advantage of making experiments reproducible; indeed, the chemical composition of field berries is highly variable, with grape varieties, vineyards and cluster positions on the vine (Zhang et al. 2008; Massart, Martinez‐Medina, and Jijakli 2015). In previous studies on SR, microorganisms were grown on generic media such as GYP for yeasts and YPM for bacteria (Barata et al. 2012; Pinto et al. 2017; Hall et al. 2018), which is different from ripening berries regarding carbon sources, namely sugars and organic acids. We evaluated the growth of the microorganism using OD data and estimated the CFUs present in the suspension through the specific calibration equations developed in this work. Turbidimetry enables rapid data generation, unlike plating methods, which do not give immediate results and are time‐consuming, especially when a large quantity of data is required (Dalgaard et al. 1994; Begot et al. 1996). To the best of our knowledge, similar studies to the present research have been previously reported regarding the growth evaluation of various genera of yeasts associated with fermentation and winemaking processes (e.g., *Torulaspora*, *Zygosaccharomyces* and *Candida*) (Catrileo, Acuña‐Fontecilla, and Godoy 2020; Mertens et al. 2011; Sipiczki 2004). The OD method was not applied to two microorganisms because the low concentration in the stock solution did not allow a clear relationship between OD data and CFUs. Finally, we obtained a good fit for the growth data as a function of temperature by using two equations frequently used in the literature (Salotti, Ji, and Rossi 2022; Fedele, Brischetto et al. 2020a).

The temperature response of the different microorganisms was in general agreement with the previous, although scarce, literature. Temperature ranges at which the two AAB grown agreed with the previously mentioned data from the BacDive metadatabase and with Holt et al. (1994), Carrascosa, Munoz, and Gonzalez (2011) and Du Toit and Pretorius (2002), except for the optimal temperature for *G. oxydans* , which was higher in our study. In agreement with Vidal‐Leira et al. (Vidal‐Leiria, Buckley, and Van Uden 1979), most of our yeasts showed sustained growth between 25°C and 35°C, and a minority had optimal temperatures at 25°C or lower. Increasing temperature requirements for *H. uvarum* , *P. fermentans* and *T. delbrueckii* were also noticed by Salvado et al. (Salvadó et al. 2011).

Based on temperature requirements for growth, we grouped the 11 microorganisms into four clusters (see Figure 6), and we assigned them to as many ecophysiological groups as physiological ecology, the study of organismal physiological response to changing environmental conditions (Konopka, 2009). One microorganism was mesophilic (with Topt higher than 35°C), one was psychrophilic (Topt lower than 15°C) and three were psychotropic (with Topt higher than 20°C to approximately 25°C) (Gounot 1986). The remaining six microorganisms showed sustained growth at 20°C to approximately 40°C but had a Topt at 30°C–35°C, lower than the typical mesophiles. We named them ‘low mesophiles’.

When we analysed the microbiome of SR‐affected grape bunches in the three bioclimates based on ecophysiological groups, we observed a clear tendency of the low mesophiles to decrease in abundance from SE to CW and NE, that is, from warm and dry to cold and humid bioclimates, and of the psychrophilic *Hanseniaspora* to increase. This result indicates that the microbiome involved in SR may change with regard to the bioclimatic conditions of the grape‐growing area. This relationship has already been defined for soil microbial communities (e.g., Costa et al. 2022; Ware et al. 2021), plant rhizospheric (e.g., Chamard et al. 2024), endospheric (e.g., Karray et al. 2020; Hansen 2018) and phyllospheric (e.g., Wang et al. 2023) microbiome. Climate was also considered a key driver of different aspects of fungal biogeography, including the global distribution of fungi, as well as the composition and diversity of fungal communities (Větrovský et al. 2019), including plant pathogenic fungi (Li et al. 2023).

That the effect of bioclimate on SR has been minimally investigated so far is surprising in comparison with other grape diseases, such as downy and powdery mildews (Khatal et al. 2023; Bendek et al. 2007), black rot (Van Niekerk et al. 2011; Onesti, González‐Domínguez, and Rossi 2017), and others (Ji et al. 2023; Savu, Tomoiaga, and Chedea 2020). The influence of temperature on the prevailing mix of bunch pathogens has also been demonstrated for rots caused by *B. cinerea* (grey mould), *Colletotrichum acutatum* (ripe rot) and *Greeneria uvicola* (bitter rot) (Steel and Greer 2006), with the former species prevailing in cold climates (having *B. cinerea* the optimal temperature at approximately 20°C–25°C and being not able to infect grape berries at > 30°C; Ciliberti et al. 2015; Latorre et al. 2002), and the latter two species being prevalent in hot climates (being able to infect berries over a range of 20°C–35°C; Steel, Greer, and Savocchia 2007).

In conclusion, our study demonstrated that bioclimate may influence the composition of microbial communities associated with the SR of grapes. However, further studies are needed to better understand the ecological requirements of the different microorganisms concerning temperature and moisture. This would be the first step in defining the ecological niches for the different SR microorganisms (Kivlin et al. 2021) to be used to understand their geographical distribution and epidemiology and improve disease control using biocontrol microorganisms (Fedele, Gonzalez Dominguez et al. 2020b). Indeed, a recent meta‐analysis revealed the potential role of biocontrol in efficient SR management (Brischetto, Rossi, and Fedele 2024c). Considering the key role of *Drosophila* flies in SR development (Hall et al. 2018; Barata et al. 2012) and the strict relationships between *Drosophila* and the SR microbiome (Brischetto, Rossi, and Fedele 2024b), the studies would also include the ecology of flies. For instance, several studies (Molon et al. 2020; Ito and Awasaki 2022) demonstrated the influence of temperature on heat flow, energy reserves, starvation survival, locomotor activity and lifespan of *Drosophila* spp.

## Author Contributions

**Chiara Brischetto:** writing – original draft, methodology, formal analysis. **Vittorio Rossi:** writing – review and editing, conceptualization, methodology, formal analysis, data curation. **Irene Salotti:** formal analysis. **Luca Languasco:** formal analysis. **Giorgia Fedele:** writing – review and editing, conceptualization, formal analysis, data curation.

## Conflicts of Interest

The authors declare no conflicts of interest.

## Supporting information


Table S1.

## Data Availability

The authors will provide the raw data supporting this article upon request.
