# Supplementary material for: Temperature Requirements Can Affect the Microbial Composition Causing Sour Rot in Grapes
Source: Environ Microbiol Rep. 2025 Jan 27;17(1):e70061. doi: 10.1111/1758-2229.70061 (PMC11772317; doi:10.1111/1758-2229.70061)
Supplement: Supplementary file 1 — Table S1. [file EMI4-17-e70061-s001.docx]

**Table 1** **supplementary material**: Calibration equations and statistics data, which described the relationship between the population densities expressed as colony forming units (CFUs) and optical density at 620nm for nine microorganisms.

| **Microorganism** | **Equation** | **R^2^** |
| --- | --- | --- |
| *Candida zemplinina* | y= 3.00E + 07x + 334508 | 0.961 |
| *Metschnikowia pulcherrima* | y= 8.00E + 07x + 415646 | 0.864 |
| *Zygoascus hellenicus* | y= 4.00E + 07x + 56540 | 0.947 |
| *Issatchenkia occidentalis* | y= 9.00E + 07x + 1.00E + 06 | 0.903 |
| *Zygosaccharomyces bailii* | y= 5.00E + 07x + 348039 | 0.939 |
| *Saccharomycopsis vini* | y= 3.00E + 07x + 585784 | 0.952 |
| *Torulospora delbrueckii* | y= 2.00E + 07x + 185464 | 0.802 |
| *Gluconobacter oxydans* | y= 5.00E + 07x – 35979 | 0.995 |
| *Acetobacter syzygii* | y= 2.08E + 08x + 3.00E + 06 | 0.968 |
